# Person‐centred care in primary care: What works for whom, how and in what circumstances?

**DOI:** 10.1111/hsc.13913

**Published:** 2022-07-21

**Authors:** Anam Ahmed, Maria E. T. C. van den Muijsenbergh, Hubertus J. M. Vrijhoef

**Affiliations:** ^1^ Panaxea b.v Amsterdam The Netherlands; ^2^ Department of Primary and Community Care Radboud University Medical Centre Nijmegen the Netherlands; ^3^ Department of Prevention and Care Pharos: Dutch Centre of Expertise on Health Disparities, Program Prevention and Care Utrecht The Netherlands; ^4^ Department of Patient & Care Maastricht University Medical Center Maastricht The Netherlands

**Keywords:** person‐centred care, primary care, primary healthcare, realist review

## Abstract

This rapid realist review aims to explain how and why person‐centred care (PCC) in primary care works (or not) among others for people with low health literacy skills and for people with a diverse ethnic and socioeconomic background, and to construct a middle‐range programme theory (PT). Peered reviewed‐ and non‐peer‐reviewed literature (Jan 2013–Feb 2021) reporting on PCC in primary care was included. Selection and appraisal of documents were based on relevance and rigour according to the Realist And Meta‐narrative Evidence Syntheses: Evolving Standards (RAMESES) criteria. Data on context, mechanisms and outcomes (CMO) were extracted. Based on the extracted data, CMO configurations were identified per source publication. Configurations containing all three constructs (CMO) were included in the PT. The middle‐range PT demonstrates that healthcare professionals (HCPs) should be trained and equipped with the knowledge and skills to communicate effectively (i.e. in easy‐to‐understand words, emphatically, checking whether the patient understands everything, listening attentively) tailored to the wishes, needs and possibilities of the patient, which may lead to higher satisfaction. This way the patient will be more involved in the care process and in the shared decision‐making process, which may result in improved concordance, and an improved treatment approach. A respectful and empathic attitude of the HCP plays an important role in establishing a strong therapeutic relationship and improved health (system) outcomes. Together with a good accessibility of care for patients, setting up a personalised care plan with all involved parties may positively affect the self‐management skills of patients. Good collaboration within the team and between different domains is desirable to ensure good care coordination. The coherence of items related to PCC in primary care should be considered to better understand its effectiveness.

**What is known about this topic?**

- Person‐centred care (PCC) is considered a core value in providing high‐quality care and therefore, increasing attention is being paid to PCC.
- The primary care setting is especially important for PCC as primary care professionals account for most of the patient care for ailments and diseases.
- As traditional research often only indicates whether PCC is more effective than standard of care, it remains unclear how PCC leads to positive results for whom and in what circumstances.

**What this paper adds?**

- This rapid realist review provides a more detailed understanding of the relationship between the *context* in which PCC in primary care is applied, the underlying *mechanisms* by which PCC in primary care does (not) work, and the *outcomes* that result from this interaction.
- Understanding of the coherence of items related to PCC in primary care is important for PCC to be effective in primary care settings.

## 1 INTRODUCTION

In healthcare, increasing attention is being paid to person‐centred care (PCC). PCC put less focus on the medical conditions and more on the unique individual with an illness or impairment (Edvardsson et al., 2017; Håkansson Eklund et al., 2019). This type of care is particularly important for people suffering from chronic diseases. Each individual is recognised as a unique person with distinct goals, needs and preferences (Håkansson Eklund et al., 2019; Kumar & Chattu, 2018; Maslow, 2013). PCC is the practice of caring for patients (and their families) in ways that are meaningful and valuable to the individual patient. It includes listening to, informing and involving patients in their care, whereby the focus is placed on the person in his personal and social context presenting the complaint or medical problem, rather than the complaint itself (Håkansson Eklund et al., 2019; WHO, 2015b). It also focuses on the social, mental, emotional and spiritual needs apart from diagnosis, physical and medical needs (Kumar & Chattu, 2018).

PCC is considered a core value in providing high‐quality (primary) healthcare (Håkansson Eklund et al., 2019; Stewart, 2005), and essential to achieving the universal health coverage goals by the World Health Organisation (WHO) (IOM, 2001; Moore et al., 2017; Pruitt & Epping‐Jordan, 2005; WHO, 2007, 2015a, 2015b). To achieve these goals, focusing on the primary care sector is essential. A strong primary care can partly contain the rising costs, for example, in the Netherlands more than 90% of the care demands are treated in primary care for only 4% of the total budget for care (Bueving, 2015; Wiegers et al., 2011). Moreover, the primary care setting is especially important for PCC as primary care physicians are the initial contact point for patients, they play an important role in ongoing healthcare, and account for the majority of patient visits for conventional illnesses (Grumbach & Bodenheimer, 2002; Schoen et al., 2006). PCC has shown positive effects on healthcare outcomes including enhanced relationships between clinicians and patients (Hamovitch et al., 2018), enhanced job satisfaction by clinicians (Sjögren et al., 2015; van der Meer et al., 2018), enhanced patient satisfaction (Edvardsson et al., 2008; Olsson et al., 2013), greater adherence to treatment improved concordance (Edvardsson et al., 2008), improved quality of life (Egan et al., 2007), and lower health care costs (Ekman et al., 2011). PCC also leads to increased self‐reliance, less anxiety, pain and depression, fewer referrals or additional patient investigation (De Silva, 2014; Eaton et al., 2015; Stewart, 2005).

Despite the global importance of PCC being generally acknowledged, the approach suffers from a lack of clarity. Traditional research, such as randomised controlled trials, meta‐analyses and systematic reviews, often only indicate whether PCC is more effective than standard of care, but does not contain information on why it was more effective and how it has led to its results given the circumstances (Dwamena et al., 2012; Maatouk‐Bürmann et al., 2016). Consequently, it remains unclear how and for whom a complex intervention such as PCC, leads to positive results and under what circumstances. Diversity in age, gender, socioeconomic status (e.g. by income, education or occupation), migration background and multi‐morbidity, is associated with large disparities in health and in quality of care (Anderson et al., 1997). Primary care research on PCC including so‐called ‘hard‐to‐reach or underserved’ groups, like non‐native speakers, migrants or ethnic minorities, people with a low educational level, or a low health literacy level, is underrepresented (van den Muijsenbergh et al., 2016), and therefore their expectations and needs are less clear, while it is known that existing care is often less suitable for them (Batterham et al., 2016; Dawson et al., 2018; Domecq et al., 2014; Schinkel et al., 2013; Schouten et al., 2007; Tierney et al., 2016).

Knowing why and how PCC leads to positive results, especially for people with low health literacy skills and for people with a diverse ethnic and socioeconomic background, is also relevant for professionals and for policymakers. To set up and implement a proactive and strong policy, it is important to have insight into the items of PCC in primary care that influence its effectiveness, considering their interrelatedness. To unravel which mechanisms are relevant for PCC in primary care and the influence of diversity on PCC, how they relate to each other, and which starting points there are to apply PCC in daily practice, a more detailed understanding of the relationship between the context in which PCC in primary care is applied and the underlying mechanisms that lead to effective PCC are needed (see ‘Methods’ section for definitions). Theretofore, the principles of realist research can be used, which focus on what works for whom, in which situation and why (Ray Pawson & Tilley, 1997). Realist research is a theory‐driven approach to review and/or evaluate complex interventions/programmes (Eaton et al., 2015; Jagosh, 2019; Pawson et al., 2005; Westhorp et al., 2011).

The objective of this study is to explain how and why PCC in primary care works (or not) among others for people with low health literacy skills and for people with a diverse ethnic and socioeconomic background, under what circumstances and to construct an overarching middle‐range programme theory.

## 2 METHODS

The review methods were established prior to the conduct of the review and there were no significant deviations from the protocol.

### 2.1 Realist approach

A rapid realist review (RRR) was conducted that followed the standard Realist And Meta‐narrative Evidence Synthesis: Evolving Standards (RAMESES) guidelines on quality and reporting (Greenhalgh et al., 2011; Wong et al., 2015). The term ‘rapid’ refers to the use of a realist approach ‘to a knowledge synthesis process and producing a product that is useful to policy makers in responding to time‐sensitive and/or emerging issues where there is limited time and resources’ (Saul et al., 2013, p. 2). The RRR focuses on explaining the relationship between the *context* in which PCC in primary care is applied, the *mechanisms* by which PCC work, and the *outcomes* that result from it. It assumes that all complex interventions have an underlying theory to explain how a particular intervention is meant to work.

### 2.2 Definitions

Several RRR terms are fundamental for understanding and assessing programmes: context, mechanisms, outcomes, context‐mechanism‐outcome configuration (CMO‐C) and programme theory (PT). These terms are briefly explained below (Jagosh et al., 2012; Ray Pawson & Tilley, 1997; Rycroft‐Malone et al., 2016; Shearn et al., 2017; Wong et al., 2013). *Context* refers to any condition that triggers and/or modifies the behaviour of a mechanism. It can include cultural norms and history of the community in which a programme is implemented, scope and the extent of existing social networks or the infrastructure in which the programme is built. They can be trust‐building processes, geographic location effects, funding sources, opportunities or constraints. *Mechanisms* describe what produces the effects of a programme and relate to causality. They are the agents of change and describe how the resources embedded in a programme influence the reasoning and action of programme ‘subjects’. They are underlying enablers, entities, processes or structures which operate in specific contexts to generate outcomes of interest. *Outcomes* are the intended and unintended results of a programme. A *CMO‐C* explains the causal relationship between a particular aspect of context, whether (or not) a mechanism of interest is triggered by it, and the outcomes produced. An *initial programme theory* is a hypothesised explanation describing how, why and for whom the complex intervention is expected to work in what circumstances. An initial PT is refined using primary or secondary evidence to a refined PT. A middle‐range PT is a theory that lies between the initial and refined PT.

### 2.3 Literature search and selection

A peer‐reviewed and a non‐peer‐reviewed literature search were conducted. The search for and the selection of literature took place in an iterative multi‐step approach, making use of a ‘purposive search’ and ‘snowball sampling’. Next to our own search, members of the steering committee were asked to share relevant key literature (see ‘Patient and public involvement’ for more information on the steering committee).

#### 2.3.1 Peer‐reviewed literature

Systematic reviews and meta‐analyses were included to provide an extensive body of broad and high‐quality evidence (Aromataris et al., 2015). The search was conducted in PubMed, Embase, Google Scholar, the Cochrane Database of Clinical Trials, and Web of Science. English and Dutch publications between January 2013 and February 2021 were included, as in older publications, most context variables were not considered presentable for current practices. Articles needed to discuss PCC in the primary care setting. Articles discussing PCC in the secondary or tertiary setting, a specific type of care (e.g. terminal care, end‐of‐life‐ care), a specific type of condition (e.g. dementia, cancer, depression) or a specific medical field (e.g. maternal health, psychiatry) were excluded. The following search terms were used (in various combinations): ‘person cent(e)red care’, ‘client cent(e)red care’, ‘people cent(e)red care’, ‘shared decision making’, ‘implementation’, ‘barrier(s)’, ‘facilitator(s)’, ‘outcome(s)’, ‘(cost‐)effectiveness’, ‘best practice’, ‘diversity’, ‘gender’, ‘vulnerable groups’, ‘illiteracy’, ‘health literacy’, ‘underserved populations’, ‘migrant(s)’, ‘ethnic minorities’ and ‘minority health’. The reference lists of eligible papers identified for the review were also searched. All articles were screened by AA and half of them by HJMV. In case of doubt, a second researcher [MvdM] was involved to make a shared decision.

#### 2.3.2 Non‐peer‐reviewed literature

The non‐peer‐reviewed literature was identified using Google. The search terms and timeframe of publications were similar to the ones in the peer‐reviewed literature search. Due to time constraints, and to capture the most relevant hits and ensuring a feasible quantity to screen, the first 15 pages (representing a total of 150 ‘best match’ results) were examined. All the selected literature was assessed on full‐text by AA and half of them by HJMV. In case of doubt, a second researcher [MvdM] was involved to make a shared decision.

### 2.4 Appraisal of documents

According to the RAMESES quality standards, the articles were appraised based on two criteria: (1) relevance (can the data contribute to theory building and/or testing?); and (2) rigour (is the method used to generate that particular piece of data credible and trustworthy?) (Wong et al., 2014). Articles were evaluated by two authors: AA evaluated all articles and HJMV half of them. In case of even a slight doubt, the researcher presented the article to the other researcher to ensure that articles were not evaluated incorrectly. Disagreements were resolved by discussion resulting in consensus.

### 2.5 Data extraction and analysis

Data on CMO of the included articles and documents were extracted by one researcher [AA], whereas HJMV extracted data from a selection of articles. Data from both the peer‐reviewed and non‐peer‐reviewed publications were considered of equal weight in the analysis.

Context items, mechanisms and outcomes were assigned to the constructs by multiple researchers independently based on the definitions of the constructs and the interpretation of the function of the items within the source publication. Disagreements about the category to which the extracted data belongs (context, mechanism or outcome) were resolved in a discussion between the researchers. Each context item, mechanism and outcome that was reported in at least six papers were included in the analysis. Given the international perspective of this study and the variety of context items, mechanisms and outcomes, we chose six papers as the minimum, realising this number is arbitrary. Then, it was examined which CMO‐C(s) could be formed based on the included context items, mechanisms and outcomes per source publication. It must be noted that since no source publication did explicitly report on the relationship between CMO and CMO‐C(s), we identified CMO‐Cs based on the items we categorised in the three constructs. Since there were also incomplete CMO‐C(s), as various source publications only reported one or two constructs (context or mechanism or outcomes), we had chosen to only include those source publications that contained all three constructs (context, mechanism and outcome). Subsequently, we analysed per outcome item (O_1_, O_2_, O_3_, etc.) which context items and mechanisms are associated with it. To report on the most described causal relation(s) per outcome item and to build a robust PT, context items and mechanisms in the CMO‐C(s) needed to be present in at least half of the included publications. Based on these CMO‐Cs, the middle‐range PT was developed describing the underlying relationships between context, mechanisms and outcomes.

### 2.6 Patient and public involvement

This study was commissioned by the National Health Care Institute, the Dutch national advisory and implementing organisation who, among others, encourages good healthcare by helping all parties involved to continually improve healthcare quality. This RRR is part of a larger study for which a steering committee was established. The ten members of the steering committee were purposively selected based on their expertise in the PCC or primary care field and were primary care practitioners, senior researchers, medical specialists, policymakers, patient's representatives (specifically concerning people with limited [health‐]literacy and a migrant background) (see Acknowledgements). Several meetings with the steering committee were held during the study (February 2018, December 2018, April 2019, and December 2019). These meetings were held with the objective to provide feedback and guidance on the methods, the interpretation of (interim) results, and providing overall advice regarding the research. Stakeholder perspectives were considered when testing and refining the PT derived from the RRR. Members of the steering committee were asked to discuss, and to indicate if the identified items on context, mechanisms and outcomes in the literature match with what they see in Dutch practice and to add anything that was possibly missing.

### 2.7 Initial programme theory

One of the objectives of realist research is to test and refine an initial PT in order to determine how, when and for whom the complex intervention will (not) work in a particular setting (Wong et al., 2013).

To formulate an initial PT on applying PCC in the primary care setting, we organised a workshop with experts of the steering committee during a kick‐off meeting (dated 28 February 2018).

During the kick‐off meeting, the study objectives and findings of the literature were shared. Participants were invited to discuss the proposed items and add relevant items. It was hypothesised that communication with the patient plays a crucial role in adequately applying PCC, especially for people with low health literacy skills or migrant background, and having sufficient time during the general practitioner (GP) consultation. The use of easy‐to‐understand language in conversations and forms (e.g. administrative, informative) when exchanging information would make the care process easier to understand for the person. It was also stated that currently the diversity aspect is not sufficiently considered when applying PCC. Also, by taking into account the context of the person, and their wishes and needs, shared decision‐making and involvement of the person would improve. Practitioners need to advance their knowledge, develop new skills and need be conscious on how they themselves apply PCC. On a more macro level, it was mentioned that PCC needs to have a more central role in medical studies at university level and that guidelines need to be adjusted for vulnerable groups, such as people with low health literacy skills. Coordination of care can be improved, as not all healthcare professionals (HCPs) involved in a patient's care process are always up‐to‐date on the progress. General practice structures and payment models were thought to limit the delivery of PCC.

## 3 RESULTS

**FIGURE 1:**
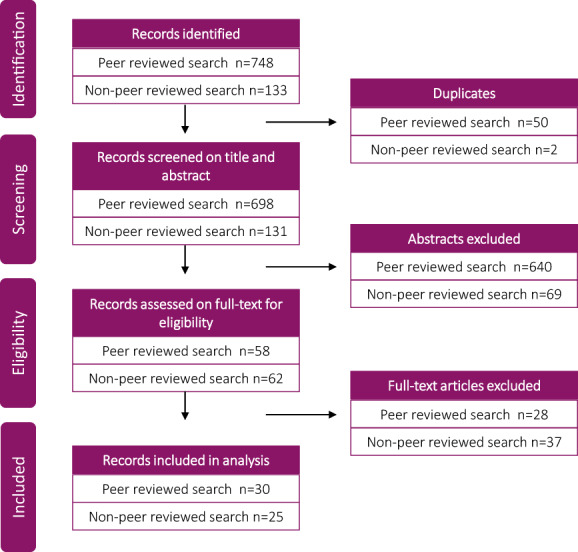
Flowchart article selection

The search strategy and inquiry through experts yielded 748 peer‐reviewed literature articles and 133 non‐peer‐reviewed articles. After duplicates were removed, 829 titles and abstracts were screened, and of these 709 publications were excluded as they did not match the inclusion criteria. The 120 remaining articles were assessed on full‐text of which 65 publications were excluded.  Fifty‐five publications were included in the analysis (Figure 1).

The design of the selected publications were:

- Seven reviews (Akseer et al., 2021; Brickley et al., 2020; Butterworth et al., 2019; Coulter et al., 2015; King & Hoppe, 2013; Lévesque et al., 2013; Louw et al., 2017);
- Thirteen systematic reviews (SR) (Derksen et al., 2013; Giusti et al., 2020; Jackson et al., 2013; Jager et al., 2019; John et al., 2020; McMillan et al., 2013; Rathert et al., 2013; Renzaho et al., 2013; Rochfort et al., 2018; Rocque & Leanza, 2015; Scholl et al., 2014; Winn et al., 2015; Winsor et al., 2013), of which one was a SR and a meta‐analysis (John et al., 2020), one was a SR and concept analysis (Scholl et al., 2014), one was a SR and qualitative meta‐synthesis (Winsor et al., 2013), and one was a SR and thematic synthesis (Jager et al., 2019)
- Seven scoping reviews (Constand et al., 2014; DeRosa et al., 2019; Filler et al., 2020; Lafontaine et al., 2020; Poitras et al., 2018; Tomaselli et al., 2020; Wildevuur & Simonse, 2015);
- Six reviews of reviews (Håkansson Eklund et al., 2019; National Voices, 2014a, 2014b, 2014c; Park et al., 2018; Sharma et al., 2015);
- One meta‐analysis (Schwartz et al., 2016);
- Three (research) articles in international journals (Lundy et al., 2015; O'Donnell et al., 2016; Smeets et al., 2020);
- Seven (research) articles in Dutch journals (de Been & van den Muijsenbergh, 2019; Ekelmans, 2020; Engelberts et al., 2018; Mutsaers & Van der Horst, 2016; van den Muijsenbergh, 2019; van der Velden, 2018; van Weel‐Baumgarten & Brouwers, 2018);
- Three guidelines (NHG, 2017; The Health Foundation, 2014, 2018);
- Two web pages (Engels, 2019; PoZoB, 2021; Van der Meulen, 2019);
- One study report (Eikelenboom, 2017; Heijmans et al., 2016);
- One white paper (Boshuizen et al., 2014);
- One information brochure (InEen., 2016);
- One PhD dissertation (Eikelenboom, 2017); and
- One commentary piece (Van den Muijsenbergh & Oostenberg, 2013).

### 3.1 Relationships between context, mechanisms and outcomes

In Table 1 an overview is provided of the items interpreted as context, mechanisms and outcomes that were extracted from the included papers with their explanation (see Appendix A for corresponding references). Items are shown in ascending order of how often they were reported in the literature. The context items concern issues related to the system‐level (macro‐level) (accessibility of care, enabling sufficient time during the consultation, and shifting from the dominant biomedical approach towards a holistic biopsychosocial approach in medicine), to the level of healthcare organisations (meso‐level) (having a good collaboration of the team, equipping HCPs with the right skillset through training, foreseeing in the required capacity, have a supporting policy in place, using information technology [IT] and e‐health initiatives), and to the level of HCPs (micro‐level) (providing patient education, setting up a personalised care plan) or the patients (having social support [networks]). Mechanisms related to behaviour of the HCP (micro‐level) (providing effective communication, for example easy to understand words, checking whether the person understands everything, listening attentively, having a holistic focus, showing respect to the person, having an open and empathic attitude, providing self‐management support, carry out shared decision‐making and provide care coordination), and of the behaviour of the person (having an active role in their care process), as well as to their interaction (establishing a therapeutic relationship). Outcomes cover health system outcomes (macro‐level), patient involvement, satisfaction of the patient, informal caregiver and/or HCP, concordance, self‐management skills, psychological outcomes, improved treatment and better health outcomes (all micro‐level).

**TABLE 1:** Reported context items (C), mechanisms (M) and outcomes (O)

Construct^a^	Explanation
Context items (C)	
Equip HCPs with the right knowledge and skills by means of professional training and education.	Training of skills concerning verbal and non‐verbal communication; ‘shared decision‐making’‐related communication; intercultural communication; tailored communication; interpersonal capacities; providing person‐centred care; to build trustful relationship with patients; empathic skills. Knowledge of medical affairs, diseases and disease processes, social and cultural differences, cultural competences. The specific knowledge and skills necessary for patients with low health literacy skills need to be included as part of the medical education.
Have a good collaboration/team	Multidisciplinary teamwork; effective interprofessional collaboration; collaboration between different domains (e.g. social domain); collaboration between patient and HCPs.
Provide patient education	Promote and provide education/educational information to patients.
Foresee in sufficient time during consultation	Lack of time is often experienced during consultations to approach patients in a holistic wat and address psychosocial problems; limitations of time affect physician‐patient relationship.
Patients having social support (networks)	Social support and social support networks, environmental support, more social support reduced sense of isolation and increased motivation and confidence.
Set up a personalised care planning	Personalised care planning in collaboration with patients (preparation, goal setting, action planning, documenting, coordinating, supporting, reviewing).
Foresee in the required capacity	Creating space for required time, people and resources and using this in a targeted manner to design person‐centred care; sufficiently equipped to accommodate the biopsychosocial needs of patients; availability of sufficient women clinicians for female patients; appropriate and effective use of healthcare resources.
Applying IT‐ and e‐health initiatives	Applying IT‐ and e‐health initiatives; providing telehealth, teleconsultations, and telemonitoring; the use of online tools and technology; developing ICT to access audiotapes of consultations and patient‐held records.
Need for shifting away from the dominance of biomedical approach in medical encounter	Too much focus on disease‐oriented and complaint‐oriented approach; too much focus on what is measurable and outcomes rather than what is necessary; evidence‐based medicine leaves limited room for patient's own considerations; current medical practice strongly based on scientific guidelines.
Accessibility of care	Offering appropriate and preferred access to care, that is care that is conveniently located for the patient (e.g. decentralised services, availability of transportation), and that can be accessed in time. It also includes accessibility to specialists or speciality services when a referral is made and (digital) access to information about care and computerised records.
Have a supporting policy in place	Policy should structurally take into account (patients with) low health literacy skills, social and cultural differences.
Mechanisms (M)	
Provide effective communication	HCPs need to provide effective communication by being compassionate, being empathetic, learning about their patients' situations through careful listening and observation, use easy language (avoid medical jargon), conveying tailored and accessible information/materials, checking the patient's understanding of the information and his or her reactions to it, deploying an interpreter.
Have a holistic focus	Understanding the whole person in addition to the presenting illness, treating the patient as a person and not a disease, nonmedical issues are considered relevant, supporting patients in their physical, psychological, social and existential needs, paying attention to the patient's life story, taking into account socio‐economic health differences.
HCPs showing respect and having an open and empathic attitude	Having an open, friendly, empathic attitude with genuine interest in and compassion for the patient. HCP needs to respect the patient's beliefs, preferences, and values, and treat them with dignity.
Patients having an active role in their care process	Engage, support, involve and empower patients to play an important active role in their care process to improve health outcomes; patient participation; involvement patient's families and informal caregivers; encourage people to use question prompts to help them interact; having family support programmes; help create awareness for the patient, explore resilience and take a step in the direction he or she wants.
Establishing a therapeutic relationship	Establishing a longitudinal doctor–patient relationship, invest in therapeutic partnership building, mutual trust.
Provide self‐management support	Provide, empower, enable self‐management (support and education) to patient.
Apply shared decision‐making	Seeking the patient's implicit or explicit involvement in the decision‐making process; exploring the patient's ideas, fears and expectations about the problem and possible treatments; providing a balanced view in the discussion of healthcare options; determine treatment goals together.
Ensure care coordination	Care that is planned and coordinated across health carers, situations, time, and across all elements of the health system; structuring service organisation to enable care continuity.
Outcomes (O)	
Health outcomes	Improvements in physical health, functional outcomes, and clinical outcomes (e.g. blood glucose levels, lung function, haemoglobin, cholesterol and blood pressure).
Patient involvement	Increased self‐efficacy, higher participation in shared decision‐making, enhanced patient autonomy.
Health system outcomes	Less referrals, less follow‐up examination, reduced emergency department visits, reduced hospital (re)admissions.
Satisfaction	Higher satisfaction of patient, informal caregiver and/or healthcare providers.
Concordance	Higher treatment and medication concordance; improved health behaviour of patient.
Self‐management skills	Improvements in self‐management skills/capabilities/activities and self‐management outcomes.
Psychological outcomes	Improvements in psychological health (e.g. depression, anxiety and distress).
Treatment approach	Improved patient‐centred treatment approach, right intensity of support, more appropriate treatment, better connection of care for people with low health literacy skills.

^a^ Items shown in ascending order of how often they were reported in the literature.

**FIGURE 2:**
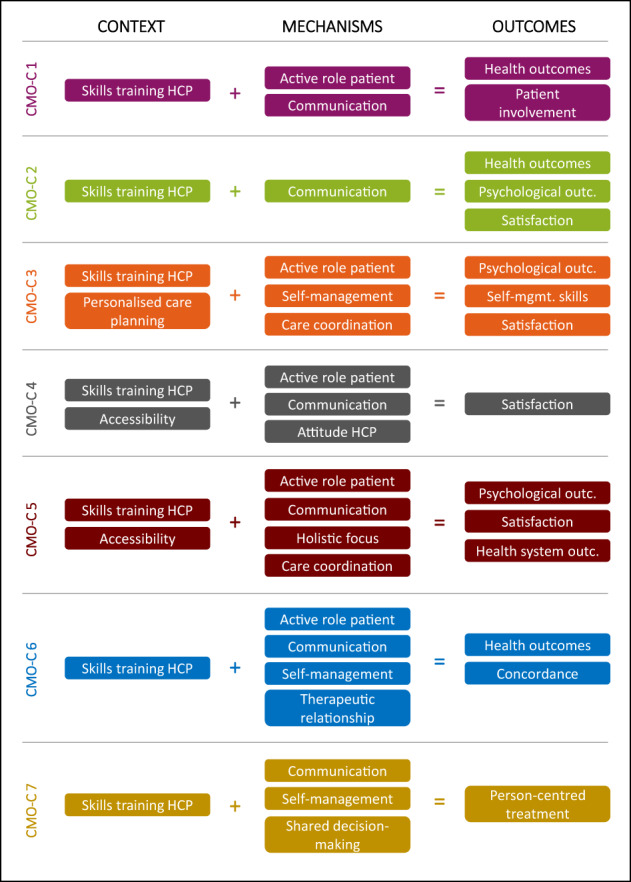
CMO‐Cs on PCC in primary care. HCP, healthcare professional, outc.: outcomes, self‐mgmt., self‐management.

Next, CMO‐Cs were aimed to be formed per source publication according to the categorisation of the items in the constructs. In Appendix B, all (complete and incomplete) CMO‐Cs as reported per source publication are shown. In Figure 2 the CMO‐configurations are shown, that contained all three constructs (i.e. context, mechanism and outcome) and are used to refine the initial PT from the workshop. For all CMO‐Cs identified, the most common context item reported in the literature was ‘skills and training HCP’ implying that HCPs need to be equipped with the knowledge and skills by means of professional training and education to perform PCC. Training of skills concern communication skills (verbal and non‐verbal, related to shared decision‐making, intercultural communication, communication tailored to the information needs and health literacy skills of the person, and teach‐back), skills to provide PCC, skills to build a trustful relationship with patients, and empathic skills. HCPs also need to have knowledge of medical diseases and disease processes, social and cultural differences and cultural competences. A second essential context item is the accessibility of care that is appropriate and in line with people's preferences, meaning care that is conveniently located for the person, affordable and that can be accessed in time. It also includes accessibility to specialist care and services when a referral is made and (digital) access to information about care and electronic patient records. Also, personalised care planning in collaboration with patients including preparation, goal setting, action planning, documenting, coordinating, supporting and reviewing, was considered an important context item of PCC. Under the influence of these context items, the following mechanisms were identified: patients (and if applicable, their informal caregivers) need to be engaged, supported, involved and empowered to play an important role in their care process to improve care outcomes. Also, HCPs need to provide effective communication by being compassionate, being empathetic, they need to learn about their patients' situations through careful listening and observation, use easy language (avoid medical jargon), convey tailored and accessible information/materials, checking the person's understanding of the information and his or her reactions to it. Moreover, providing and empowering self‐management (support and education) to the patient was considered an important mechanism. Important outcomes of PCC in primary care, as the result of the interaction between context items and mechanisms, are improved health outcomes, psychological outcomes and health system outcomes, improved self‐management skills, improved concordance, higher satisfaction of the patient, informal caregivers and/or healthcare providers, more involvement of the patient in his/her care process, and a more adequate person‐centred treatment whereby the right intensity of support is offered to the patient.

### 3.2 Middle‐range PT

It was found in both the initial PT and the RRR that communication (M) tailored to the needs and health literacy skills of the patient plays an important role in, among others in the extent to which patients are and feel involved in their care process (M), and also in the shared decision‐making process (M). To communicate effectively and to acquire other necessary skills (M), HCPs need to be trained and educated (C) to have a PCC approach during treatment (M) instead of a biomedical, disease‐oriented approach (M). HCPs should be provided with sufficient time (C) to discuss the wishes and preferences of patients (M). If several HCPs are involved in the care process, good collaboration within the team (C) and between different domains (C) is desirable to ensure good care coordination (M). Also, supporting policies (C) help to address the importance of PCC.

Based on the RRR, the initial PT can be further complemented: respect and attitude of the HCP (M) play an important role in establishing a strong therapeutic relationship (M). Providing patient education (C) and setting up a personalised care plan (C) together with patient positively affects the self‐management skills (O). Patients' social support networks (C) also help to improve the patients' health (O). In addition, having sufficient capacity (C), offering access to appropriate and preferred care (C), and providing IT and telephone initiatives (C) play a key role in practicing PCC in primary care.

There were several items that were not observed in the RRR but were mentioned by experts when establishing the initial PT. These concerns take the diversity aspect more into account when applying PCC, PCC having a more central role in medical studies, and having general practice structures and payment models in place that facilitate PCC in primary care.

## 4 DISCUSSION

### 4.1 Principal findings

This study aims to explain how and why PCC in primary care works (or not) among others for people with low health literacy skills and for people with a diverse ethnic and socioeconomic background, under what circumstances and to construct a middle‐range programme theory. In this RRR, the middle‐range theory demonstrates that HCPs should be trained and equipped with the knowledge and skills to communicate effectively (in easy‐to‐understand words, emphatically, listening attentively, checking whether the patient understands everything) tailored to the wishes, needs and possibilities of the patient, which may lead to higher satisfaction. This way patients will be and feel more involved in their care process and in the shared decision‐making process, which may result in improved concordance, and an improved treatment approach. A respectful and empathic attitude of the HCP plays an important role in establishing a strong therapeutic relationship causing improved health (system) outcomes. Together with a good accessibility of care for patients, setting up a personalised care plan with all involved parties may positively affect the self‐management skills of patients. Good collaboration within the team and between different domains is desirable to ensure good care coordination.

Two items (i.e. the need for more attention to diversity in patients when practicing PCC and more teaching of PCC in medical education) that were not observed in the RRR, but mentioned by experts in the initial PT, may be party included in other context items found in the RRR. Concerning diversity, the RRR identifies the context items ‘having a holistic focus’ and ‘HCP respecting the patient's beliefs, preferences and values’, which implies understanding the whole person in addition to the presenting illness, treating the patient as a person and not a disease, non‐medical issues being considered relevant, supporting patients in their physical, psychological, social and existential needs, paying attention to the patient's life story, taking into account ethnic and socio‐economic health differences. This indicates that if HCPs work in a person‐centred way, one automatically would have to pay attention to the diversity aspect. Also, the item PCC having a more central role in medical studies, which was included in the initial PT but not found in the RRR may be counterbalanced by the context item ‘equipping HCPs with the knowledge and skills by means of professional training and education’. Regarding the identified context items, mechanisms and outcomes, it was observed that context items interpreted by us were reported on system‐level (macro‐level), the level of healthcare organisations (meso‐level), and at the level of HCPS and patients (micro‐level), whereas mechanisms were only reported on micro‐level, and outcomes on macro‐level and micro‐level.

### 4.2 Strengths and limitations

To the best of our knowledge, this is the first RRR on the effectiveness of person‐centred care in primary care, providing insight into the complex interplay of context, mechanisms and outcomes. Also, the coherence of items in relation to PCC in primary care has not been reported before. This study is in line with the Realist And Meta‐narrative Evidence Syntheses: Evolving Standards (RAMESES) quality and publication standards (Wong et al., 2014; Wong et al., 2015), which focus on the objectives of Realist Review, understanding and applying a realist principle, realist review design, data collection methods, involving key stakeholders, data analysis and reporting a realist review. A methodological limitation, inherent to realist research, is that the instructions for performing a Realist Review are only partially crystallised. This can be both limiting and reinforcing, since during the process of reflection and decision‐making, researchers can make adjustments to the realist constructs, but cannot estimate whether these adjustments will bring out the best result. A second limitation to consider, also inherent to realist research, is the lack of conceptual clarity of the constructs context (J. Greenhalgh & Manzano, 2021; Renmans et al., 2020) and mechanisms (Lacouture et al., 2015; Lemire et al., 2019), which makes assigning items to the different constructs difficult (Marchal et al., 2012). Our interpretation of items was based on the definition of context and mechanisms (see methods section), and information provided in the source publications, which was often limited. Also, several context items (e.g. providing patient education) and mechanisms (e.g. applying shared decision‐making) can be considered independent interventions, as the source publication itself did not label the items as context or mechanisms, but we interpreted the items as context or mechanism. To ensure that items were assigned to the constructs correctly, multiple researchers independently examined the interpretation of the function of the items within the publication as closely as possible. Lastly, many of the included studies did not have complete data to construct the most optimal CMO configurations. This may have to do with the emphasis placed on outcome data in many studies, and to a lesser extent on mechanisms of action and context. A large part of the CMO configurations was incomplete only containing one or two constructs. To paint a valid picture of the most reported CMO configurations, incomplete CMO configurations were excluded meaning that a lot of information was lost.

### 4.3 Comparison with prior work

A previous realist synthesis which aimed to elicit an initial PT of how multispecialty community providers can achieve their outcomes has found strong evidence on multidisciplinary teams being an important mechanism provided that the teams include the relevant professions (Sheaff et al., 2018). Causal relations were also found with the uses and effects of health information technology (HIT) and care planning for individual patients (Sheaff et al., 2018). Contrary to what we found, they also reported on organisational culture, interorganisational network management, planned referral networks and the diversion of patients from inpatient to primary care (Sheaff et al., 2018). In line with our findings, a synthesis on person‐centred models reported that patients (and their families) and caregivers valued three key features of PCC, namely strong communication skills among HCPs to facilitate shared decision‐making and positive patient‐provider relationships; having a certain level of control on health decisions and treatment plan(s); and patients being treated as an individual with their own preferences and needs, rather than simply as a patient with a disease. Also, team‐based primary care was desirable due to the benefits of better collaboration among HCPs. HCPs educating patients on their illness was observed as a way to enhance PCC at the system, organisational and/or provider level (Cheng et al., 2018). A framework on PCC approaches mentioned the core elements of communication (including communication between personnel at all levels in an organisation) and relationship‐building skills as key players (Fagan et al., 2017).

### 4.4 Recommendations

Further research needs to be conducted concerning the extent to which the items identified in this RRR are currently collectively being applied in practice. Ideally, to make the PT more robust, more studies with data on all CMO‐items in the CMO configurations should be available to validate findings and the PT. This way, one can also analyse which combinations of CMO configurations concerning PCC in primary care do not take place and therefore, when PCC does not work.

To be able to more accurately assess the items influencing PCC for understudied groups like ethnic minorities, or people with low (health) literacy skills, more data on health and healthcare use of these groups are necessary. To this end, registration of ethnicity and educational level should be included in databases on health and healthcare use. We also recommend to promote PCC in practice through actions on macro ‐ meso and micro level: at policy level we recommend: the development and implementation of quality indicators for PCC, comprehensible communication and accessibility of care (also for people with limited health literacy skills); setting requirements for training of HCPs and for guideline development, stimulating the development of integrated multidisciplinary care standards for multimorbidity instead of disease‐specific standards; setting requirements for e‐Health/IT activities ensuring that IT‐systems in different settings can be integrated to enable collaboration and coordination between HCPs; and facilitating flexible consultation time and adjustment of care intensity to patient needs as well as interprofessional collaboration between healthcare and social care. On an organisation level (meso‐level) PCC needs to be included in the vision and policy and be discussed with all employees how PCC can be achieved within their own practice; patients need to be involved in the design and organisation of practice and care; good accessibility of the practice needs to be ensured; interprofessional training of all HCPs needs to be stimulated and facilitated. On micro‐level HCPs should educate themselves (in PCC, self‐reflection, understandable communication) and apply what they have learned. They should approach each patient with an open respectful attitude, focused on the patient's questions, problems, wishes and values in addition to on the illness or medical complaint.

## 5 CONCLUSION

This RRR provides insight into the complex interplay of context, mechanisms and outcomes concerning PCC in primary care. The coherence of items related to PCC in primary care should be considered to better understand its effectiveness. HCPs should be trained and stimulated to communicate empathically, understandably and culturally sensitive, focused on the wishes, needs and possibilities of the patient, so that self‐management can be realised as much as possible. In addition to requiring knowledge and skills, a good accessibility to care, as well as setting up personalised care plans with the active involvement of the patient (and his/her family) is required, so that these can result in improved health (system) outcomes, improved concordance, higher satisfaction and a more adequate person‐centred treatment.

## AUTHORS' CONTRIBUTION

AA: methods design, search strategy, selection and appraisal of papers, data collection, data extraction, data analysis, interpretation of data, design and writing of the manuscript. METCM: interpretation of data, regularly reviewing the work, providing feedback on manuscript, manuscript final approval. HJMV: concept and design overall study, appraisal of papers, data analysis, interpretation of data, regularly reviewing the work, providing feedback on manuscript, manuscript final approval.

## FUNDING INFORMATION

Panaxea B.V. received funding for the larger study from the Dutch National Health Care Institute (grant number: 2017055441). No funding was received for the preparation of this manuscript. The funding party had no role in the study design; in the collection, analysis and interpretation of data; in the writing of the manuscript; and in the decision to submit the article for publication.

## CONFLICT OF INTEREST

The authors declare that they have no competing interests.

## ETHICS APPROVAL AND CONSENT TO PARTICIPATE

Not applicable.

## CONSENT FOR PUBLICATION

Not applicable.

## Supporting information


Appendix A

Appendix B

## Data Availability

The data that support the findings of this study are available from the corresponding author upon reasonable request.
