# Supplementary material for: Person‐centred care in primary care: What works for whom, how and in what circumstances?
Source: Health Soc Care Community. 2022 Jul 21;30(6):e3328–41. doi: 10.1111/hsc.13913 (PMC10083933; doi:10.1111/hsc.13913)
Supplement: Supplementary file 1 — Appendix A [file HSC-30-e3328-s002.pdf]

Appendix A. References of context items, mechanisms, and outcomes

Construct	References
**Context items (C)**	
Equip HCPs with the right knowledge and skills by means of professional training and education.	Brickley et al., 2020; Butterworth et al., 2019; de Been & van den Muijsenbergh, 2019; Derksen, Bensing, & Lagro-Janssen, 2013; Ekelmans, 2020; Filler, Jameel, & Gagliardi, 2020; Giusti et al., 2020; Heijmans, Zwikker, van der Heide, & Rademakers, 2016; Jager et al., 2019; King & Hoppe, 2013; Lafontaine, Bourgault, Girard, & Ellefsen, 2020; Lévesque, Hovey, & Bedos, 2013; Mutsaers, 2016; O'Donnell et al., 2016; Poitras, Maltais, Bestard-Denommé, Stewart, & Fortin, 2018; Rathert, Wyrwich, & Boren, 2013; Renzaho, Romios, Crock, & Sønderlund, 2013; Rochfort et al., 2018; Rocque & Leanza, 2015; Scholl, Zill, Härter, & Dirmaier, 2014; Sharma, Bamford, & Dodman, 2015; Smeets, Kroese, Ruwaard, Hameleers, & Elissen, 2020; The Health Foundation, 2018; Van den Muijsenbergh, 2013; van der Velden, 2018; van Weel-Baumgarten & Brouwers, 2018; Winsor et al., 2013
Have a good collaboration/team	Boshuizen, 2014; Constand, MacDermid, Dal Bello-Haas, & Law, 2014; DeRosa et al., 2019; Filler et al., 2020; Giusti et al., 2020; InEen, 2016; Jackson et al., 2013; Jager et al., 2019; John, Jani, Peters, Agho, & Tannous, 2020; Louw, Marcus, & Hugo, 2017; Mutsaers, 2016; Poitras et al., 2018; Scholl et al., 2014; Sharma et al., 2015; Smeets et al., 2020
Provide patient education	Boshuizen, 2014; DeRosa et al., 2019; Eikelenboom, 2017; John et al., 2020; Lafontaine et al., 2020; National Voices, 2014a, 2014c; Park et al., 2018; Scholl et al., 2014; Winn, Ozanne, & Sepucha, 2015
Foresee in sufficient time during consultation	Akseer et al., 2021; Brickley et al., 2020; Butterworth et al., 2019; de Been & van den Muijsenbergh, 2019; Giusti et al., 2020; Mutsaers, 2016; Rocque & Leanza, 2015; Sharma et al., 2015; Smeets et al., 2020
Patients having social support (networks)	Eikelenboom, 2017; Jager et al., 2019; Lafontaine et al., 2020; O'Donnell et al., 2016; Park et al., 2018; Smeets et al., 2020; Tomaselli, Buttigieg, Rosano, Cassar, & Grima, 2020; Winsor et al., 2013

Set up a personalised care planning	Coulter et al., 2015; InEen, 2016; NHG, 2017; Poitras et al., 2018; Rathert et al., 2013; Smeets et al., 2020; Wildevuur & Simonse, 2015
Foresee in the required capacity	Constand et al., 2014; Filler et al., 2020; Giusti et al., 2020; InEen, 2016; Jager et al., 2019; National Voices, 2014a; Smeets et al., 2020
Applying IT- and e-health initiatives	Boshuizen, 2014; InEen, 2016; Jager et al., 2019; National Voices, 2014a, 2014b, 2014c; Smeets et al., 2020; Wildevuur & Simonse, 2015; Winsor et al., 2013
Need for shifting away from the dominance of biomedical approach in medical encounter	Ekelmans, 2020; Engelberts, 2018; Mutsaers, 2016; Poitras et al., 2018; PoZoB, 2021; Rocque & Leanza, 2015
Accessibility of care	de Been & van den Muijsenbergh, 2019; Heijmans et al., 2016; Jackson et al., 2013; O'Donnell et al., 2016; Park et al., 2018; Scholl et al., 2014
Have a supporting policy in place	Boshuizen, 2014; de Been & van den Muijsenbergh, 2019; Heijmans et al., 2016; O'Donnell et al., 2016; Smeets et al., 2020; Van der Meulen, 2019
Mechanisms (M)	
Provide effective communication	Akseer et al., 2021; Boshuizen, 2014; Butterworth et al., 2019; Constand et al., 2014; de Been & van den Muijsenbergh, 2019; Derksen et al., 2013; Engels, 2019; Filler et al., 2020; Giusti et al., 2020; Heijmans et al., 2016; Jager et al., 2019; King & Hoppe, 2013; Muijsenbergh, 2019; National Voices, 2014a, 2014b; O'Donnell et al., 2016; Park et al., 2018; PoZoB, 2021; Rathert et al., 2013; Rocque & Leanza, 2015; Scholl et al., 2014; Sharma et al., 2015; Smeets et al., 2020; Van der Meulen, 2019; van der Velden, 2018; van Weel-Baumgarten & Brouwers, 2018; Wildevuur & Simonse, 2015
Have a holistic focus	Brickley et al., 2020; de Been & van den Muijsenbergh, 2019; Filler et al., 2020; Giusti et al., 2020; Håkansson Eklund et al., 2019; Jackson et al., 2013; Lafontaine et al., 2020; Louw et al., 2017; McMillan et al., 2013; Muijsenbergh, 2019; O'Donnell et al., 2016; Park et al., 2018; Poitras et al., 2018; PoZoB, 2021; Rocque & Leanza, 2015; Scholl et al., 2014;

	Schwartz, Weiner, Binns-Calvey, & Weaver, 2016; Sharma et al., 2015; The Health Foundation, 2018; Tomaselli et al., 2020; Van den Muijsenbergh, 2013; Van der Meulen, 2019
HCPs showing respect and having an open and empathic attitude	Akseer et al., 2021; Brickley et al., 2020; DeRosa et al., 2019; Ekelmans, 2020; Filler et al., 2020; Giusti et al., 2020; Håkansson Eklund et al., 2019; Lafontaine et al., 2020; Lundy et al., 2015; Mutsaers, 2016; Rathert et al., 2013; Rocque & Leanza, 2015; Sharma et al., 2015; Tomaselli et al., 2020; Van den Muijsenbergh, 2013; van der Velden, 2018; van Weel-Baumgarten & Brouwers, 2018
Patients having an active role in their care process	Akseer et al., 2021; Butterworth et al., 2019; Coulter et al., 2015; DeRosa et al., 2019; Eikelenboom, 2017; Ekelmans, 2020; Giusti et al., 2020; McMillan et al., 2013; National Voices, 2014c; Park et al., 2018; PoZoB, 2021; Rathert et al., 2013; Scholl et al., 2014; Sharma et al., 2015; The Health Foundation, 2014, 2018; Tomaselli et al., 2020; van Weel-Baumgarten & Brouwers, 2018; Wildevuur & Simonse, 2015
Establishing a therapeutic relationship	Akseer et al., 2021; Brickley et al., 2020; Constand et al., 2014; Filler et al., 2020; Giusti et al., 2020; Håkansson Eklund et al., 2019; InEen, 2016; Lafontaine et al., 2020; Louw et al., 2017; Muijsenbergh, 2019; Mutsaers, 2016; Park et al., 2018; Scholl et al., 2014; Sharma et al., 2015; The Health Foundation, 2018; van der Velden, 2018; van Weel-Baumgarten & Brouwers, 2018; Winsor et al., 2013
Providing self-management support	Boshuizen, 2014; Butterworth et al., 2019; Coulter et al., 2015; Eikelenboom, 2017; Filler et al., 2020; Heijmans et al., 2016; InEen, 2016; Jager et al., 2019; John et al., 2020; National Voices, 2014b; Poitras et al., 2018; Rochfort et al., 2018; The Health Foundation, 2014, 2018; Winsor et al., 2013
Apply shared decision-making	Brickley et al., 2020; Butterworth et al., 2019; Coulter et al., 2015; Eikelenboom, 2017; Ekelmans, 2020; Engelberts, 2018; Giusti et al., 2020; Håkansson Eklund et al., 2019; InEen, 2016; Lafontaine et al., 2020; Mutsaers, 2016; Park et al., 2018; Sharma et al., 2015; Smeets et al., 2020; The Health Foundation, 2014
Ensure care coordination	Giusti et al., 2020; Håkansson Eklund et al., 2019; Jackson et al., 2013; John et al., 2020; O'Donnell et al., 2016; Park et al., 2018; Poitras et al., 2018; PoZoB, 2021; Scholl et al., 2014; The Health Foundation, 2014; Winsor et al., 2013

Outcomes (O)	
Health outcomes	Coulter et al., 2015; de Been & van den Muijsenbergh, 2019; Derksen et al., 2013; John et al., 2020; King & Hoppe, 2013; McMillan et al., 2013; Muijsenbergh, 2019; Park et al., 2018; Rathert et al., 2013; Renzaho et al., 2013; The Health Foundation, 2018; Tomaselli et al., 2020; Van den Muijsenbergh, 2013; van Weel-Baumgarten & Brouwers, 2018; Wildevuur & Simonse, 2015
Patient involvement	de Been & van den Muijsenbergh, 2019; Derksen et al., 2013; DeRosa et al., 2019; Muijsenbergh, 2019; National Voices, 2014a, 2014c; PoZoB, 2021; Rochfort et al., 2018; Rocque & Leanza, 2015; Winn et al., 2015
Health system outcomes	Butterworth et al., 2019; de Been & van den Muijsenbergh, 2019; Jackson et al., 2013; John et al., 2020; McMillan et al., 2013; O'Donnell et al., 2016; Park et al., 2018; Van den Muijsenbergh, 2013; van Weel-Baumgarten & Brouwers, 2018; Wildevuur & Simonse, 2015
Satisfaction	Brickley et al., 2020; Derksen et al., 2013; King & Hoppe, 2013; Lafontaine et al., 2020; McMillan et al., 2013; Park et al., 2018; Rathert et al., 2013; Rocque & Leanza, 2015; The Health Foundation, 2014
Concordance	Brickley et al., 2020; Coulter et al., 2015; King & Hoppe, 2013; National Voices, 2014a, 2014b; Rochfort et al., 2018; Rocque & Leanza, 2015; The Health Foundation, 2018; van Weel-Baumgarten & Brouwers, 2018
Self-management skills	Coulter et al., 2015; John et al., 2020; National Voices, 2014b; Park et al., 2018; Rathert et al., 2013; Rochfort et al., 2018; Winsor et al., 2013
Psychological outcomes	Butterworth et al., 2019; Coulter et al., 2015; de Been & van den Muijsenbergh, 2019; Derksen et al., 2013; John et al., 2020; Muijsenbergh, 2019
Improved treatment	Boshuizen, 2014; Eikelenboom, 2017; Heijmans et al., 2016; InEen, 2016; McMillan et al., 2013; O'Donnell et al., 2016; Park et al., 2018; Rocque & Leanza, 2015; van der Velden, 2018; van Weel-Baumgarten & Brouwers, 2018

References:

Akseer, R., Connolly, M., Cosby, J., Frost, G., Kanagarajah, R. R., & Lim, S.-H. E. (2021). Clinician–patient relationships after two decades of a paradigm of patient-centered care. *International Journal of Healthcare Management, 14*(3), 888-897.

Boshuizen, D., Engels, J., Versleijen, M., Vlek, H., Rebel, M., & Driessen, S. (2014). White paper – Hoe maak je een succes van persoonsgerichte zorg? In. Utrecht, The Netherlands.

Brickley, B., Sladdin, I., Williams, L. T., Morgan, M., Ross, A., Trigger, K., & Ball, L. (2020). A new model of patient-centred care for general practitioners: results of an integrative review. *Fam Pract, 37*(2), 154-172. doi:10.1093/fampra/cmz063

Butterworth, J. E., Hays, R., McDonagh, S. T., Richards, S. H., Bower, P., & Campbell, J. (2019). Interventions for involving older patients with multi-morbidity in decision-making during primary care consultations. *Cochrane Database Syst Rev, 2019*(10). doi:10.1002/14651858.CD013124.pub2

Constand, M. K., MacDermid, J. C., Dal Bello-Haas, V., & Law, M. (2014). Scoping review of patient-centered care approaches in healthcare. *BMC Health Serv Res, 14*, 271. doi:10.1186/1472-6963-14-271

Coulter, A., Entwistle, V. A., Eccles, A., Ryan, S., Shepperd, S., & Perera, R. (2015). Personalised care planning for adults with chronic or long-term health conditions. *Cochrane Database Syst Rev, 2015*(3), Cd010523. doi:10.1002/14651858.CD010523.pub2

de Been, M., & van den Muijsenbergh, M. (2019). persoons-gerichte, integrale zorg. *TVZ-Verpleegkunde in praktijk en wetenschap, 129*(4), 16-18.

Derksen, F., Bensing, J., & Lagro-Janssen, A. (2013). Effectiveness of empathy in general practice: a systematic review. *Br J Gen Pract, 63*(606), e76-84. doi:10.3399/bjgp13X660814

DeRosa, A. P., Baltich Nelson, B., Delgado, D., Mages, K. C., Martin, L., & Stribling, J. C. (2019). Involvement of information professionals in patient- and family-centered care initiatives: a scoping review. *J Med Libr Assoc, 107*(3), 314-322. doi:10.5195/jmla.2019.652

Eikelenboom, N. (2017). *Personalised self-management support in primary care: one size does not fit all.* [Sl: sn],

Ekelmans, N. (2020). Persoonsgerichte zorg leidt tot betere uitkomsten op lange (re) termijn. *Nederlands Tijdschrift voor Diabetologie, 18*(3), 38-40.

Engelberts, I., Schermer, M., & Prins, A. . (2018). Een goed gesprek is de beste persoonsgerichte zorg. *Medisch Contact, 30-31*, 18-20.

Engels, J. (2019). Zelfmanagement en gezondheidsvaardigheden. Retrieved from https://www.zorgvoorbeter.nl/persoonsgerichte-zorg/gezondheidsvaardigheden

Filler, T., Jameel, B., & Gagliardi, A. R. (2020). Barriers and facilitators of patient centered care for immigrant and refugee women: a scoping review. *BMC Public Health, 20*(1), 1013. doi:10.1186/s12889-020-09159-6

Giusti, A., Nkhoma, K., Petrus, R., Petersen, I., Gwyther, L., Farrant, L., . . . Harding, R. (2020). The empirical evidence underpinning the concept and practice of person-centred care for serious illness: a systematic review. *BMJ global health, 5*(12), e003330.

Håkansson Eklund, J., Holmström, I. K., Kumlin, T., Kaminsky, E., Skoglund, K., Höglander, J., . . . Summer Meranius, M. (2019). "Same same or different?" A review of reviews of person-centered and patient-centered care. *Patient Educ Couns, 102*(1), 3-11. doi:10.1016/j.pec.2018.08.029

Heijmans, M., Zwikker, H., van der Heide, I., & Rademakers, J. (2016). NIVEL Kennisvraag 2016: Zorg op maat. *Hoe kunnen we de zorg beter laten aansluiten bij mensen met lage gezondheidsvaardigheden*.

InEen. (2016). Special: persoonsgerichte zorg. In. Utrecht, The Netherlands.

Jackson, G. L., Powers, B. J., Chatterjee, R., Bettger, J. P., Kemper, A. R., Hasselblad, V., . . . Williams, J. W. (2013). The patient centered medical home. A systematic review. *Ann Intern Med, 158*(3), 169-178. doi:10.7326/0003-4819-158-3-201302050-00579

Jager, M., de Zeeuw, J., Tullius, J., Papa, R., Giammarchi, C., Whittal, A., & de Winter, A. F. (2019). Patient Perspectives to Inform a Health Literacy Educational Program: A Systematic Review and Thematic Synthesis of Qualitative Studies. *Int J Environ Res Public Health, 16*(21). doi:10.3390/ijerph16214300

John, J. R., Jani, H., Peters, K., Agho, K., & Tannous, W. K. (2020). The Effectiveness of Patient-Centred Medical Home-Based Models of Care versus Standard Primary Care in Chronic Disease Management: A Systematic Review and Meta-Analysis of Randomised and Non-Randomised Controlled Trials. *Int J Environ Res Public Health, 17*(18). doi:10.3390/ijerph17186886

King, A., & Hoppe, R. B. (2013). "Best practice" for patient-centered communication: a narrative review. *J Grad Med Educ, 5*(3), 385-393. doi:10.4300/jgme-d-13-00072.1

Lafontaine, S., Bourgault, P., Girard, A., & Ellefsen, E. (2020). Dimensions, application, and outcomes of person-centered self-management interventions for those living with type 2 diabetes: a scoping review. *Patient Educ Couns, 103*(10), 1961-1982.

Lévesque, M., Hovey, R., & Bedos, C. (2013). Advancing patient-centered care through transformative educational leadership: a critical review of health care professional preparation for patient-centered care. *Journal of Healthcare Leadership, 5*, 35-46.

Louw, J. M., Marcus, T. S., & Hugo, J. F. M. (2017). Patient- or person-centred practice in medicine? - A review of concepts. *Afr J Prim Health Care Fam Med, 9*(1), e1-e7. doi:10.4102/phcfm.v9i1.1455

Lundy, J.-M., Bikker, A., Higgins, M., Watt, G. C., Little, P., Humphries, G. M., & Mercer, S. W. (2015). General practitioners' patient-centredness and responses to patients' emotional cues and concerns: relationships with perceived empathy in areas of high and low socioeconomic deprivation. *Journal of Compassionate Health Care, 2*(1), 2. doi:10.1186/s40639-015-0011-6

McMillan, S. S., Kendall, E., Sav, A., King, M. A., Whitty, J. A., Kelly, F., & Wheeler, A. J. (2013). Patient-centered approaches to health care: a systematic review of randomized controlled trials. *Med Care Res Rev, 70*(6), 567-596. doi:10.1177/1077558713496318

Muijsenbergh, v. d. (2019). Gezondheidsverschillen vragen om persoonsgerichte, integrale zorg door eerstelijns-en publieke gezondheidszorg. *Tijdschrift voor gezondheidswetenschappen, 97*(1), 36-39. doi:10.1007/s12508-019-0223-9

Mutsaers, I., & Van der Horst, H. . (2016). Maak van persoonsgerichte zorg geen ideologie. *Medisch Contact, 43*, 37-39.

National Voices. (2014a). *Improving information and understanding.* . Retrieved from London, United Kingdom:

National Voices. (2014b). *Supporting self-management*. Retrieved from London, United Kingdom:

National Voices. (2014c). *Supporting shared decision-making*. Retrieved from London, United Kingdom:

NHG. (2017). Handreiking - Gezamenlijke besluitvorming over doelen en zorgafspraken. . In. Utrecht, the Netherlands.

O'Donnell, C. A., Burns, N., Mair, F. S., Dowrick, C., Clissmann, C., van den Muijsenbergh, M., . . . MacFarlane, A. (2016). Reducing the health care burden for marginalised migrants: The potential role for primary care in Europe. *Health Policy, 120*(5), 495-508. doi:10.1016/j.healthpol.2016.03.012

Park, M., Giap, T. T., Lee, M., Jeong, H., Jeong, M., & Go, Y. (2018). Patient- and family-centered care interventions for improving the quality of health care: A review of systematic reviews. *Int J Nurs Stud, 87*, 69-83. doi:10.1016/j.ijnurstu.2018.07.006

Poitras, M. E., Maltais, M. E., Bestard-Denommé, L., Stewart, M., & Fortin, M. (2018). What are the effective elements in patient-centered and multimorbidity care? A scoping review. *BMC Health Serv Res, 18*(1), 446. doi:10.1186/s12913-018-3213-8

PoZoB. (2021). Aan de slag met persoonsgerichte zorg. . Retrieved from https://www.pozob.nl/persoonsgerichtezorg/aan-de-slag-met-persoonsgerichte-zorg/

Rathert, C., Wyrwich, M. D., & Boren, S. A. (2013). Patient-centered care and outcomes: a systematic review of the literature. *Med Care Res Rev, 70*(4), 351-379. doi:10.1177/1077558712465774

Renzaho, A. M., Romios, P., Crock, C., & Sønderlund, A. L. (2013). The effectiveness of cultural competence programs in ethnic minority patient-centered health care--a systematic review of the literature. *Int J Qual Health Care, 25*(3), 261-269. doi:10.1093/intqhc/mzt006

Rochfort, A., Beirne, S., Doran, G., Patton, P., Gensichen, J., Kunnamo, I., . . . Collins, C. (2018). Does patient self-management education of primary care professionals improve patient outcomes: a systematic review. *BMC Fam Pract, 19*(1), 163. doi:10.1186/s12875-018-0847-x

Rocque, R., & Leanza, Y. (2015). A Systematic Review of Patients' Experiences in Communicating with Primary Care Physicians: Intercultural Encounters and a Balance between Vulnerability and Integrity. *PLoS One, 10*(10), e0139577. doi:10.1371/journal.pone.0139577

Scholl, I., Zill, J. M., Härter, M., & Dirmaier, J. (2014). An integrative model of patient-centeredness - a systematic review and concept analysis. *PLoS One, 9*(9), e107828. doi:10.1371/journal.pone.0107828

Schwartz, A., Weiner, S. J., Binns-Calvey, A., & Weaver, F. M. (2016). Providers contextualise care more often when they discover patient context by asking: meta-analysis of three primary data sets. *BMJ Qual Saf, 25*(3), 159-163. doi:10.1136/bmjqs-2015-004283

Sharma, T., Bamford, M., & Dodman, D. (2015). Person-centred care: an overview of reviews. *Contemp Nurse, 51*(2-3), 107-120. doi:10.1080/10376178.2016.1150192

Smeets, R. G., Kroese, M. E., Ruwaard, D., Hameleers, N., & Elissen, A. M. (2020). Person-centred and efficient care delivery for high-need, high-cost patients: primary care professionals' experiences. *BMC Fam Pract, 21*(1), 1-9. doi:10.1186/s12875-020-01172-3

The Health Foundation. (2014). *Ideas into practice: person-centered care in practice*. Retrieved from London, United Kingdom:

The Health Foundation. (2018). *Person-centred care made simple - What everyone should know about person-centred care.* . Retrieved from London, United Kingdom:

Tomaselli, G., Buttigieg, S. C., Rosano, A., Cassar, M., & Grima, G. (2020). Person-Centered Care From a Relational Ethics Perspective for the Delivery of High Quality and Safe Healthcare: A Scoping Review. *Front Public Health, 8*, 44. doi:10.3389/fpubh.2020.00044

Van den Muijsenbergh, M., & Oostenberg, E.H. (2013). Patiëntgericht én cultureel competent Goede zorg voor allochtone patiënten vereist specifieke competenties. *Ned Tijdschr Geneeskd., 157*(A5612).

Van der Meulen, M. (2019). Interview - Aandacht voor cultuursensitieve zorg hard nodig. . Retrieved from https://www.vilans.nl/artikelen/interview-aandacht-voor-cultuursensitieve-zorg-hard-nodig

van der Velden, J. H. (2018). Persoonsgerichte zorg bij mensen met een migratieachtergrond. *Bijblijven, 34*(3), 207-217.

van Weel-Baumgarten, E. M., & Brouwers, M. (2018). Persoonsgerichte communicatie–wat is dat en wat levert het op? *Bijblijven, 34*(3), 218-227.

Wildevuur, S. E., & Simonse, L. W. (2015). Information and communication technology-enabled person-centered care for the "big five" chronic conditions: scoping review. *J Med Internet Res, 17*(3), e77. doi:10.2196/jmir.3687

Winn, K., Ozanne, E., & Sepucha, K. (2015). Measuring patient-centered care: An updated systematic review of how studies define and report concordance between patients' preferences and medical treatments. *Patient Educ Couns, 98*(7), 811-821. doi:10.1016/j.pec.2015.03.012

Winsor, S., Smith, A., Vanstone, M., Giacomini, M., Brundisini, F. K., & DeJean, D. (2013). Experiences of patient-centredness with specialized community-based care: a systematic review and qualitative meta-synthesis. *Ont Health Technol Assess Ser, 13*(17), 1-33.
