# Supplementary material for: Person‐centred care in primary care: What works for whom, how and in what circumstances?
Source: Health Soc Care Community. 2022 Jul 21;30(6):e3328–41. doi: 10.1111/hsc.13913 (PMC10083933; doi:10.1111/hsc.13913)
Supplement: Supplementary file 2 — Appendix B [file HSC-30-e3328-s001.pdf]

Appendix B. CMO-configurations per source publication

Overview context, mechanisms, and outcomes (CMO)

Context items	Mechanisms	Outcomes
C_1: Equip HCPs with the right knowledge and skills by means of professional training and education.	M_1: Patients having an active role in their care process	O_1: Health outcomes
C_2: Provide patient education	M_2: Provide effective communication	O_2: Psychological outcomes
C_3: Have a supporting policy in place	M_3: Providing self-management support	O_3: Self-management skills
C_4: Patients having social support (networks)	M_4: Apply shared decision-making	O_4: Concordance
C_5: Accessibility of care	M_5: HCPs showing respect and having an open and empathic attitude	O_5: Satisfaction
C_6: Foresee in the required capacity	M_6: Have a holistic focus	O_6: Patient involvement
C_7: Applying IT- and e-health initiatives	M_7: Ensure care coordination	O_7: Treatment approach
C_8: Foresee in sufficient time during consultation	M_8: Establishing a therapeutic relationship	O_8: Health system outcomes
C_9: Need for shifting away from the dominance of biomedical approach in medical encounter		
C_{10}: Have a good collaboration/team		
C_{11}: Set up a personalised care planning		

Overview CMO-configurations per source publication

CMO-configurations			Source (first author, year)
C8	M1, M2, M5, M8	-	Akseer et al. (2021)
C2, C3, C7, C10, C11	M2, M3	O7	Boshuizen et al. (2014)
C1, C8	M1, M5, M6, M8	O4, O5, O6	Brickley et al. (2020)
C1, C8	M1, M2, M3, M4	O4, O5	Butterworth et al. (2019)
C6, C10	M1, M2, M5, M8	-	Constand et al. (2014)
C11	M1, M3, M4	O1, O2, O3, O4	Coulter et al. (2015)
C1, C3, C7	M2, M6	O1, O2, O6	De Been et al. (2019)
C2, C10	M1, M5	-	DeRosa et al. (2019)
C1	M2	O1, O2, O5, O6	Derksen et al. (2013)
C1, C9	M1, M4, M5	-	Ekelmans (2020)

C4	M3, M4	O7	Eikelenboom (2017)
C9	M1, M4	-	Engelberts et al. (2018)
-	M2	-	Engels (2019)
C1, C6, C10	M2, M4, M5, M6, M8	-	Filler et al. (2020)
C1, C6, C8, C10	M1, M2, M4, M5, M6, M7, M8	-	Giusti et al. (2020)
-	M4, M5, M6, M7, M8	-	Håkansson Eklund et al. (2019)
C3, C5, C1	M2, M3	O7	Heijmans et al. (2016)
C6, C7, C11	M3, M4, M7	O7	InEen (2016)
C10, C5	M6, M7	O8	Jackson et al. (2013)
C1, C4, C6, C7, C10	M3	-	Jager et al. (2019)
C2, C10	M3, M7	O2, O3, O8	John et al. (2020)
C1	M2, M4, M8	O1, O4, O5	King et al. (2013)
C1, C2, C4	M1, M4, M5, M6	O5, O7	Lafontaine et al. (2020)
C1	-	-	Levesque et al. (2013)
C10	M5, M6, M8	-	Louw et al. (2017)
-	M5	-	Lundy et al. (2015)
-	M1, M5, M6	O1, O5	McMilan et al. (2013)
C1, C8, C9, C10	M4, M5, M8	-	Mutsaers et al. (2016)
C7	M2, M3	O4	National voices (2014a)
C7	M1	-	National voices (2014b)
C2, C6, C7	M2	-	National voices (2014c)
C11	-	-	NHG (2017)
C1, C3, C4, C5	M2, M6, M7	O8	O'Donnell et al. (2016)
C1, C2, C5, C11	M1, M4, M6, M7	Patients: O3, O5, O8; family members: O2, O5; HCPs: O2	Park et al. (2018)
C1, C9, C10	M3, M6, M7	-	Poitras et al. (2018)
C9	M1, M6, M7	O6	PoZoB (2021)
C1, C11	M1, M2, M5	O1, O3, O5	Rathert et al. (2013)
C1	M1	-	Renzaho et al. (2013)
C1	M1, M3	O3, O4, O6	Rochfort et al. (2018)
C1, C8, C9	M1, M2, M5	O5, O6	Rocqueet al. (2015)

C1, C2, C5, C9, C10	M1, M2, M7, M8	-	Scholl et al. (2014)
-	-	-	Schwartz et al. (2016)
C1, C10	M1, M2, M6, M8	-	Sharma et al. (2015)
C1, C6, C7, C8, C10, C11	M2, M4	-	Smeets et al. (2020)
-	M1, M3, M4, M7	O5	The Health Foundation (2014)
C1	M1, M3, M5, M8	O1, O4	The Health Foundation (2018)
C4	M1, M5, M6	O1	Tomaselli et al. (2020)
C1	M5, M6	O1, O5	Van den Muijsenbergh et al. (2013)
C1	M2, M8	O1, O2, O6	Van den Muijsenbergh (2019)
C3	M2, M6	-	Van der Meulen (2019)
C1	M2, M5, M8	O7	Van der Velden (2018)
C1	M1, M2, M5, M8	O1, O4, O8	Van Weel-Baumgarten et al. (2018)
C2, C7, C11	-	O1, O8	Wildevuur et al. (2015)
C2	-	O6	Winn et al. (2015)
C1, C4, C7	M3, M7, M8	O3	Winsor et al. (2013)
